# Association between the recognition of muscle mass and exercise habits or eating behaviors in female college students

**DOI:** 10.1038/s41598-021-04518-8

**Published:** 2022-01-12

**Authors:** Tomoki Mase, Kumiko Ohara, Katsumasa Momoi, Harunobu Nakamura

**Affiliations:** 1 Faculty of Human Development and Education, Kyoto Women’s University, 35 Kitahiyoshi-cho, Imakumano, Higashiyama, Kyoto, Kyoto 605-8501 Japan; 2 Department of Public Health, Kindai University Faculty of Medicine, 377-2 Oono-Higashi, Osaka-Sayama, Osaka 589-8511 Japan; 3 Faculty of Health and Welfare, Tokushima Bunri University, 180 Nishihama-hoji, Yamashiro-cho, Tokushima, Tokushima 770-8514 Japan; 4 Graduate School of Human Development and Environment, Kobe University, 3-11 Tsurukabuto, Nada, Kobe, Hyogo 657-8501 Japan

**Keywords:** Human behaviour, Nutrition, Weight management

## Abstract

This study aimed to examine the association between muscle mass and perception of body shape, desired body shape, physical strength, exercise habits, and eating behaviors. Height, weight, and body composition in 270 female university students were measured. The questionnaire on body shape perception, desired body shape, dieting experience, current, and past exercise habits, exercise preference, and eating behaviors were administered. The analysis of covariance with body fat mass as the covariate found that the skeletal muscle index (SMI) was different among each group on each of body perception or desired body shape (all, *p* < 0.001). In the post hoc test on body shape perception, the SMI in “obese” was significantly more than that in “slim” (*p* < 0.001) and “normal” (*p* < 0.001). In the desired body shape, the SMI in “become thin” was more than that in “maintain as current shape” (*p* < 0.001). Further, a significant difference was found among the categories of diet experience, with body fat mass as the covariate. In the post hoc test, the SMI in “yes” was more than that in “no” (*p* < 0.001). These results indicate that not only body fat mass but skeletal muscle mass drives young females’ desire for thinness even with exercise advantages.

## Introduction

Skeletal muscle plays an important role in physical activity and posture maintenance^1, 2^, and maintaining proper muscle mass is acquired through physical activity and health maintenance^3, 4^. Particularly, weakness, fracture, fall, and joint disease, which are common in the elderly, are related to sarcopenia, a condition in which the skeletal muscle mass and strength decrease with aging and muscle weakness or physical function decline^5, 6^. Moreover, sarcopenia was reported to not only cause falls and fractures but also increase the morbidity and mortality risk of various diseases, such as diabetes mellitus and cardiovascular diseases^7–9^. Therefore, maintaining a healthy musculoskeletal is important to maintain physical function, avoid the risk of sarcopenia, and lead an independent and high-quality life. Until now, age-related muscle loss and muscle weakness were more pronounced in the 40 s and 50s^10, 11^, and many studies were reported on the prevention and improvement of sarcopenia in middle-aged and elderly people^12–14^. Contrarily, the amount of muscle mass acquired by humans peaks in their 20 s and gradually decreases with age^10, 15^. Controlling muscle weakness is important, which is also important to prevent sarcopenia.

In Japan, it has been reported in recent years that young women tend to lose weight over time^16^. According to the National Health and Nutrition Survey, the proportion of underweight women in all women and women in their twenties was 8.4% and 13.1% in 1980, 8.4% and 18.3% in 1990, and 10.3% and 24.5 in 2000, respectively. The percentage has remarkably increased especially in the 20 s, and in recent years, the percentage of thin people in their 20 s has been in the 20% range^17^. In addition, the body mass index (BMI) is low in persons with low muscle mass^18, 19^, that is, muscle mass above average is not acquired in an underweight woman. Further, the number of normal weight people with both low muscle mass and high-fat percentage, so-called “hidden obesity,” is also increasing^20^. Particularly, the report shows that Asians have more hidden obesity than white people, which has become one of the concerns^21–23^.

Thus, health problems related to physique and body composition are of concern for young women^22, 24^, and women with low muscle mass are considered to be at a greater risk of developing sarcopenia in the future. Those who need support and care are mostly female^25^, and the number of patients with sarcopenia is mostly female^26^. Therefore, acquired muscle mass in young women should be examined from the viewpoint of sarcopenia prevention; however, reports are few on this subject. Additionally, even many of normal weight young women have a desire for thinness. If these women select a dietary restriction to become thin, the normal weight women also have a potential risk of sarcopenia in the future. Thus, those who are normal weight young women should be also included for investigation of sarcopenia prevention.

The exercise was reported to be an important factor to gain muscle mass^27^. The prevalence of sarcopenia has also been reported to be reduced by continuing intense exercise during puberty^28, 29^. In addition, reports suggest that women’s exercise is associated with other habits and behaviors of daily life, such as smoking, alcohol consumption, or diets^30, 31^. However, they focus on the amount of energy and nutrients from exercise and dietary intake and do not necessarily mention the behavioral factors behind exercise and dietary intake.

Therefore, this study aimed to examine the relationship between muscle mass and exercise habits or eating behaviors that could affect muscle mass gain in female college students and examine the relationship among muscle mass and bone mass, fat mass, and physical strength.

## Methods

### Participants

The study was conducted during university classes in 2015 and 2016. The participants were 293 female students who attended the classes in the university in Kyoto City, Japan. Participants did not receive remuneration. The questionnaire, anthropometry measurement, body composition, and physical fitness test were conducted. The participants were 18–24 years old. The questionnaires were administered to 293 students. Of the 293 questionnaires, 270 questionnaires contained valid responses. Thus, the response rate, calculated by dividing the number of valid responses by the number of delivered questionnaires, was 92.2% (n = 270, 18–24 years). All procedures performed in studies involving human participants were following the ethical standards of the institutional and national research committee and with the 1964 Helsinki declaration and its later amendments or comparable ethical standards. The study protocol was approved by the Research Ethics Committee of Kyoto Women’s University. Informed consent was obtained from all individual participants included in the study.

### Measurements of body composition

Bioelectrical impedance analysis (BIA) (Inner Scan 50VBC-622, Tanita, Tokyo) was used to measure body composition. The participants did not consume alcoholic beverages the night before. On the day of measurement, measurements were not done immediately after food or drinking consumption or immediately after exercise, but after at least 1 h after meal or exercise. BMI was calculated by body weight (kg) divided by the square of height (m). Skeletal muscle mass index (SMI) (kg/m^2^) was calculated by extremity muscle mass (kg) divided by the square of height (m) according to the method by Baumgartner et al.^32^.

### Questionnaire

The questionnaire was conducted on perception of body shape, desire for a body shape, dieting experience, current exercise habit, past exercise habit, exercise preference, and eating behaviors, which were referred to previous reports^24, 33^. On the perception of body shape, the participants were asked, “How do you perceive your body shape?” The responses included “slim,” “a little slim,” “normal,” and “obese.” Furthermore, desired body shape was asked, and the responses included “be of large body size,” “maintain the current shape,” or “become thin.” Participants who selected the response “become thin” were then asked about their reasons for the desire to be thin. The responses included “for health” and “for beauty.” Dieting experience was asked and the response included “yes” or “no.” Participants who responded “yes” to their dieting experience were also asked about their main method of dieting (“exercise,” “reduce meals,” or “skip snacks”). Then, “reduce meals” and “skip snacks” were categorized into 1 group named “diet restriction.” Current exercise habit was asked and the responses included “seldom,” “a few times per month,” “a few times per week,” or “usually.” We categorized “seldom” and “a few times per month” into “not habitual” and “a few times per week” and “usually” into “habitual.” Past exercise habits included questions about “habitual playing in childhood,” “exercise in elementary school,” “participation of exercise club in junior high school,” or “participation of exercise club in high school.” The responses for “habitual playing in childhood” include “seldom,” “a few times per month,” “a few times per week,” or “usually.” We categorized “seldom” and “a few times per month” into “not habitual” and “a few times per week” and “usually” into “habitual.” The responses for “exercise in elementary school” include “seldom,” “a few times per month,” “a few times per week,” or “usually.” We categorized “seldom” and “a few times per month” into “not habitual” and “a few times per week” and “usually” into “habitual.” The responses for “participation of exercise club in junior high school” or “participation of exercise club in high school” include “yes” or “no.” “Current exercise preference” or “Exercise preference in childhood” was asked and the responses include “like” or “dislike.”

The Japanese version of the Eating Attitude Test-26 (EAT-26) was used to assess eating behavior; its validity and responsibility were validated in previous studies^34, 35^. EAT-26 is a 26-item self-rated questionnaire that is divided into 3 subscales: dieting (13 items), bulimia and food preoccupation (6 items), and oral control (7 items)^36^. The items are rated on a 6-point Likert scale: never, rarely, sometimes, often, usually, and always. According to the original study by Garner et al.^36^, the responses were quantified by assigning a score of 0 for never, rarely, or sometimes; 1 for often; 2 for usually; and 3 for always. Participants with a total EAT-26 score of ≥ 20 were designated as “seriously disturbed.”

#### Physical fitness test

The new physical fitness test of the Japanese Ministry of Education and Science was conducted following the guidelines for the new physical fitness test set by the Ministry of Education, Culture, Sports, Science, and Technology^37^, which included “grip strength,” “sit-up,” “sit-and-reach,” “side-to-side jump,” and “standing broad jump.” On grip strength, the participant held the Smedley handgrip dynamometer with the pointer pointing outward, naturally opened both legs to the left and right in an upright posture, naturally lowered his arms, and grasped the grip strength meter as hard as he could without touching his body or clothes and without shaking the grip strength system. They performed the exercise twice, alternately, on each side, and averaged the records of the best one on each side. On sit-up, the participant was lying on the mat, holding his hands lightly, crossing his arms in front of his chest, and keeping his knees at 90°. The assistant held the participant’s knees and fixed them. At the signal of “Start,” the upper body was raised from the supine position until both elbows touched both thighs, and immediately returned to the supine position. This was repeated for 30 s. This exercise was performed once and the number of times the upper body was raised was recorded. On sit-and-reach, the participant puts both legs between the long-seat anteflexion meter, takes a long sitting posture, stretches the back muscles, and puts them back and hips on the wall. With the participant's shoulders wide, the participant's palms were placed on the measuring instrument, the participant's elbows were extended, and slowly bent forward to prevent the participant's knees from bending, and after maximally bending forward, the distance traveled by the measuring instrument was recorded. The record of the person who may carry it out twice was adopted. On side-to-side-jump, the participant stood across the centerline of three parallel lines drawn at intervals of 100 cm, and at the signal “Start,” the participant side-stepped until he crossed or stepped on either the left or right line, then returned to the centerline, and did the same for the line on the opposite side. One point was given for each line passed on both sides. It was carried out twice and a better record was adopted. On standing broad jump, the participant stood with both feet slightly open so that the toes were in line with the front edge of the stepping line, stepped forward with both feet at the same time, and measured the distance in a straight line connecting the position closest to the stepping line and the front edge of the stepping line. A long-seat anteflexion meter (T.K.K. 5412, Takei Scientific Instruments Co., Ltd. Niigata, Japan) was used to evaluate “sit-and-reach.” A Smedley handgrip dynamometer (T.K.K. 5401, Takei Scientific Instruments Co., Ltd. Niigata, Japan) was used to measure the grip strength.

### Data analysis

Participants were divided into tertiles by SMI, and the first, second, and third tertiles were categorized into Low, Middle, and High groups, respectively. One-way analysis of variance (ANOVA) was used to evaluate the differences among the 3 groups, and the Bonferroni test was used for the post hoc test. The Jonckheere–Terpstra test was used to evaluate the trend analysis among the three groups. A Chi-square test was used to evaluate the response rate in cross-tabulation. The Mantel–Haenszel test was used to evaluate the trend analysis in cross-tabulation. Statistical analysis of body perception, a desired body shape, and diet experience by ANOVA and analysis of covariance (ANCOVA), adjusted by body fat mass (kg), was also performed. Regarding desired body shape, the number of participants in the “be of large body size” group was two; thus, ANCOVA was tested by excluding the “be of large body size” group. Statistical Package for the Social Sciences^®^ 22.0 for Windows (IBM, Armonk, NY, USA) was used for statistical analyses. *P*-values equal to 0.05 were considered statistically significant.

## Results

### Participant characteristics

The participants’ physical characteristics are presented in Table 1. The average height, body weight, and BMI were 158.2 ± 5.2 cm, 54.2 ± 7.7 kg, and 21.6 ± 2.8 kg/m^2^, respectively. The SMI, an index of muscle mass, was 6.3 ± 0.8 kg/m^2^.

**Table 1:** Characteristics of subjects.

	Mean ± SD	Range
Age (year)	19.0 ± 1.1	(18.0–24.0)
Height (cm)	158.2 ± 5.2	(142.0–169.5)
Body weight (kg)	54.2 ± 7.7	(39.3–81.5)
Body mass index (kg/m^2^)	21.6 ± 2.8	(16.2–31.7)
Body fat mass (kg)	16.1 ± 5.1	(6.3–36.7)
Body fat percentage (%)	29.1 ± 5.3	(13.8–48.9)
Lean body mass (kg)	38.1 ± 3.5	(29.9–50.3)
Whole body muscle mass (kg)	36.1 ± 3.1	(28.7–46.3)
Limb skeletal muscle mass (kg)	15.7 ± 2.0	(12.1–22.8)
Whole body bone mineral contents (kg)	2.2 ± 0.3	(1.5–3.3)
Skeletal muscle mass index (kg/m^2^)	6.3 ± 0.8	(5.0–9.5)

### Differences between groups

The physical characteristics of the 3 groups are presented in Table 2. According to one-way ANOVA, a significant difference was found among the 3 groups in all items. In multiple comparisons, height was lower in the High group than that in the Low group (*p* < 0.05). Bodyweight, BMI, lean body mass (LBM), whole body muscle mass, estimated bone mineral contents, and SMI were significantly higher in the Middle group than in the Low group (*p* < 0.05) and significantly higher in the High group than in the other two groups (*p* < 0.05). The body fat mass was significantly higher in the Middle group or the High group than in the Low group (*p* < 0.05), and the body fat percentage was significantly higher in the Middle group than in the Low group (*p* < 0.05). Trend analysis indicated that all items, except for height, showed higher values as muscle mass increased.

**Table 2:** Comparison of anthropometry and body composition among three groups.

	Low SMI group (n = 90)	Middle SMI group (n = 91)	High SMI group (n = 89)	*p *value	*p* for trend
Height (cm)	159.3 ± 4.9	157.8 ± 5.1	157.4 ± 5.3*	0.032	0.022
Body weight (kg)	49.9 ± 4.9	54.6 ± 7.2*	58.0 ± 8.3*^†^	< 0.001	< 0.001
Body mass index (kg/m^2^)	19.7 ± 1.4	21.9 ± 2.2*	23.4 ± 3.1*^†^	< 0.001	< 0.001
Body fat mass (kg)	14.1 ± 3.2	16.6 ± 4.9*	17.6 ± 6.2*	< 0.001	< 0.001
Body fat percentage (%)	27.9 ± 4.1	29.9 ± 5.0*	29.6 ± 6.4	0.022	0.052
Lean body mass (kg)	35.9 ± 2.4	38.0 ± 3.0*	40.4 ± 3.5*^†^	< 0.001	< 0.001
Whole body muscle mass (kg)	34.2 ± 2.2	36.1 ± 2.7*	38.2 ± 3.1*^†^	< 0.001	< 0.001
Limb skeletal muscle mass (kg)	14.1 ± 0.9	15.4 ± 1.0	17.8 ± 1.8	< 0.001	< 0.001
Whole body bone mineral contents (kg)	2.0 ± 0.2	2.2 ± 0.3*	2.4 ± 0.3*^†^	< 0.001	< 0.001
Skeletal muscle index (kg/m^2^)	5.5 ± 0.2	6.2 ± 0.2*	7.2 ± 0.6*^†^	< 0.001	< 0.001

Data are mean ± standard deviation.

Jonckheere–Terpstra test was used for trend analysis.

Analysis of variance was used for comparison among three groups. Bonferroni method was used for post-hoc analysis.

SMI: skeletal muscle mass index.

*Significantly different from low SMI group (*p* < 0.05).

^†^Significantly different from middle SMI group (*p* < 0.05).

### Muscle mass and physical fitness

The physical fitness test results are presented in Table 3. The physical fitness test results indicated higher values as the muscle mass increased in all the measurement items, and the one-way ANOVA revealed a significant difference between the three groups in all items, except for sit-and-reach. After multiple comparisons, the High group exhibited significantly higher values in the side-to-side jump than that of the Low group (*p* < 0.05). The High group exhibited significantly higher values in the grip strength, sit-up and standing broad jump than the other two groups (*p* < 0.05). Trend analysis by the Jonckheere–Terpstra test revealed significance in all items, except for sit-and-reach.

**Table 3:** Results of physical fitness tests in three groups.

	Low SMI group (n = 90)	Middle SMI group (n = 91)	High SMI group (n = 89)	*p* value	*p* for trend
Grip strength (kg)	23.9 ± 5.0	24.5 ± 4.7	26.6 ± 4.9*^†^	0.001	0.001
Sit-up (number of times)	19.0 ± 6.4	19.5 ± 5.3	22.8 ± 6.1*^†^	0.001	0.001
Sit-and-reach (cm)	41.7 ± 9.3	42.4 ± 11.0	44.0 ± 8.9	0.375	0.167
Side-to-side jump (number of times)	43.8 ± 6.4	45.4 ± 5.6	47.2 ± 6.3*	0.009	0.006
Standing broad jump (cm)	154.4 ± 21.8	153.9 ± 21.2	163.8 ± 20.3*^†^	0.016	0.023

Data are mean ± standard deviation.

Jonckheere–Terpstra test was used for trend analysis.

Analysis of variance was used for comparison among three groups, and Bonferroni method was used for post-hoc test.

SMI: skeletal muscle mass index.

*Significantly different from low SMI group (*p* < 0.05).

^†^Significantly different from middle SMI group (*p* < 0.05).

### Tendency analysis

The relationship among body perception; desired body shape; reason for slimming; diet experience; method of diet; current exercise habit; playing in early childhood; exercise experience in the elementary, junior high, or high school days; exercise preference in childhood; current exercise preference; and SMI groups is presented in Table 4. Significant differences were found in the items of perception of body shape, desired body shape, diet experience, and exercise preference in childhood among the three groups (*p* < 0.05). Trend analysis revealed significant differences in perception of body shape, desired body shape, diet experience, and exercise preference in childhood.

**Table 4:** Comparison of body shape, diet, exercise among three groups.

	Low SMI group (n %)	Middle SMI group (n %)	High SMI group (n %)	Total % (n)	*p* value	*p* for trend
**Body shape perception**						
Obese	54.4 (49)	79.1 (72)	87.7 (78)	73.7 (199)	< 0.001	< 0.001
Normal	32.2 (29)	17.6 (16)	11.2 (10)	20.4 (55)		
Slim	13.3 (12)	3.3 (3)	1.1 (1)	5.9 (16)		
**Desired body shape**						
Be of a large body size	2.2 (2)	0.0 (0)	0.0 (0)	0.7 (2)	< 0.001	< 0.001
To maintain	33.3 (30)	11.0 (10)	5.6 (5)	16.7 (45)		
Become thin	64.5 (58)	89.0 (81)	94.4 (84)	82.6 (223)		
**Reason to be thin**						
For health	19.0 (11)	24.7 (20)	28.6 (24)	24.7 (55)	0.427	0.196
For beauty	81.0 (47)	75.3 (61)	71.4 (60)	75.3 (168)		
**Diet experience**						
Yes	46.7 (42)	72.5 (66)	83.1 (74)	67.4 (182)	< 0.001	< 0.001
No	53.3 (48)	27.5 (25)	16.9 (15)	32.6 (88)		
**Method for diet**						
Exercise	23.8 (10)	18.2 (12)	29.7 (22)	24.2 (44)	0.281	0.345
Diet restriction	76.2 (32)	81.8 (54)	70.3 (52)	75.8 (138)		
**Current exercise habit**						
No	92.2 (83)	85.7 (78)	88.8 (79)	88.9 (240)	0.379	0.460
Yes	7.8 (7)	14.3 (13)	11.2 (10)	11.1 (30)		
**Habitual playing in childhood**						
No	30.0 (27)	37.4 (34)	29.7 (29)	33.3 (90)	0.566	0.712
Yes	70.0 (63)	62.6 (57)	67.4 (60)	66.7 (180)		
**Exercise habit in elementary school**						
No	28.9 (26)	31.9 (29)	16.9 (15)	25.9 (70)	0.052	0.067
Yes	71.1 (64)	68.1 (62)	83.1 (74)	74.1 (200)		
**Exercise habit in junior high school**						
No	40.0 (36)	44.0 (40)	31.5 (28)	38.5 (104)	0.213	0.243
Yes	60.0 (54)	56.0 (51)	68.5 (61)	61.5 (166)		
**Exercise habit in high school**						
No	72.2 (65)	78.0 (71)	68.5 (61)	73.0 (197)	0.352	0.583
Yes	27.8 (25)	22.0 (20)	31.5 (28)	27.0 (73)		
**Exercise preference in childhood**						
Like	66.7 (60)	64.8 (59)	85.4 (76)	72.2 (195)	0.003	0.005
Dislike	33.3 (30)	35.2 (32)	14.6 (13)	27.8 (75)		
**Current exercise preference**						
Like	64.4 (58)	65.9 (60)	77.5 (69)	69.3 (187)	0.116	0.059
Dislike	35.6 (32)	34.1 (31)	22.5 (20)	30.7 (83)		

Chi-square test was used for comparison among groups.

SMI: skeletal muscle mass index.

Mantel–Haenszel test was used for trend test.

Those who answered that they were fat were 54.4%, 79.1%, and 87.7% in the Low, Middle, and High groups, respectively, indicating an increasing trend with increasing muscle mass. More than 80% of the respondents stated their desire to gain body shape, with 64.5%, 89.0%, and 94.4% in the Low, Middle, and High groups, respectively, saying that they want to lose weight. The percentage of those who answered “for health” was 19.0%, 24.7%, and 28.6% in the Low, Middle, and High groups, respectively, which indicated an increasing trend with increasing muscle mass. Regarding dieting experience, approximately 70% of the total respondents who answered “have dieting experience” accounted for 46.7%, 72.5%, and 83.1 in the Low, Middle, and High groups, respectively. Furthermore, a clear difference was not found in the ratio of answers in the main dieting methods tried by respondents with dieting experience; however, “stop snacking” and “reduce meals” were implemented. In the Low, Middle, and High groups, > 70% were in each case, and approximately 80% of those had experienced dieting.

A clear difference was not found in the ratio of responses for current exercise habits. For past exercise habits, no clear difference was found in the ratio of responses regarding the presence or absence of habitual exercise or play outside of preschool and kindergarten during childhood and the presence or absence of participation in an athletic club in junior high and high school. Notably, 71.1%, 68.1%, and 83.1% of respondents in the Low, Middle, and High groups, respectively, answered that they had exercised in elementary school. In addition, 66.7%, 64.8%, and 85.4% in the Low, Middle, and High groups, respectively, tend to increase muscle mass as those who liked or disliked exercise during childhood. Similarly, those who answered “like” about current exercise likes and dislikes were 64.4%, 65.9%, and 77.5% in the Low, Middle, and High groups, respectively, which showed an increasing trend with the increased muscle mass. Trend analysis by the Mantel–Haenszel test revealed a significance in the items of body shape perception, desired body shape, diet experience, and exercise preference in childhood.

### Muscle mass and eating behavior

The results of EAT-26 are presented in Table 5. The average value and standard deviation of the total score of participants in the EAT-26 by the replacement score were 7.6 ± 6.3. In addition, the percentage of those who have a total score of 20 or more, who were judged to have a strong tendency to eat abnormally, is 6 (2.2%), 4 (1.5%), and 6 (2.2%) in the Low, Middle, and High groups, respectively (total = 16 [5.9%]). Comparing the total score and factor score of EAT-26, the one-way ANOVA revealed a significant difference among the three groups in the first factor that is, “dieting.” Significantly higher values were observed (*p* < 0.05). Trend analysis by the Jonckheere–Terpstra test revealed significance in “dieting” (factor I).

**Table 5:** Comparison of EAT-26 among groups.

	Low SMI group(n = 90)	Middle SMI group (n = 91)	High SMI group (n = 89)	*p-value*	*p* for trend
EAT-26 Total score	7.2 ± 6.6	8.0 ± 6.2	7.8 ± 6.1	0.662	0.293
Dieting (factor I)	3.3 ± 4.1	4.9 ± 4.5*	5.1 ± 4.2*	0.009	0.002
Bulimia and food preoccupation (factor II)	1.8 ± 2.8	1.3 ± 1.8	1.4 ± 2.2	0.233	0.364
Oral control (factor III)	2.0 ± 2.6	1.8 ± 2.1	1.4 ± 1.7	0.103	0.352

Data are mean ± standard deviation.

EAT-26: Eating Attitude Test-26, SMI: skeletal muscle mass index.

Jonckheere–Terpstra test was used for trend analysis.

Analysis of variance was used for comparison among three groups, and Bonferroni method was used for post-hoc analysis.

*Significantly different from low SMI group (*p* < 0.05).

### Muscle mass and body perception, body shape desire, diet experience

The difference in SMI among categories of body perception, desired body shape, or diet experience evaluated by ANOVA and ANCOVA is presented in Table 6. A significant difference was found among the categories of body perception, with body fat mass as the covariate. In the post hoc test, SMI in “obese” was significantly more than in “slim” (*p* < 0.001) and “normal” (*p* < 0.001). Significant differences were also found among the categories of a desired body shape, with body fat mass as the covariate. In the post hoc test, SMI in “become thin” was more than “maintain as current shape” (*p* < 0.001). Furthermore, a significant difference was found among the categories of diet experience, with body fat mass as the covariate. In the post hoc test, SMI was more in “yes” than “no” (*p* < 0.001).

**Table 6:** Results of analysis of covariance.

	n	SMI	ANCOVA *p*
**Body perception**			
Slim	16	5.6 ± 0.4	< 0.001
Normal	55	5.9 ± 0.6	
Obese	199	6.4 ± 0.8*^†^	
**Desired body shape**			
To maintain	45	5.8 ± 0.7	< 0.001
Become thin	223	6.4 ± 0.8^‡^	
**Diet experience**			
No	88	6.0 ± 0.6	< 0.001
Yes	182	6.4 ± 0.7^§^	

Analysis of covariance was used for each comparison of body perception, desired body shape, or diet experience. Bonferroni method or unpaired *t* test was used for post-hoc test.

ANCOVA: analysis of covariance, SMI: skeletal muscle mass index.

*Significantly different from “slim” (*p* < 0.05).

^†^Significantly different from “normal” (*p* < 0.05).

^‡^Significantly different from “To maintain” (*p* < 0.05).

^§^Significantly different from “No” (*p* < 0.05).

## Discussion

This study examined the relationship between muscle mass and eating behavior, exercise habits, physical fitness, body shape recognition, desired body shape, and diet experience in female university students.

According to one-way ANOVA, the relationship between SMI and anthropometry was significant. All items, except for the percentage of body fat, were positively significant by trend analysis. Bone mass was reported to be related to the skeletal muscle mass^38^, which is mediated by insulin-like growth factor 1^39–41^. The weight per unit volume is heavy in the muscle compared with the body fat. Thus, the more muscle mass the participants have, the more BMI they have.

Physique, such as height and weight, affects muscle mass^15^. In the definition and diagnostic criteria of sarcopenia, SMI, which is the height-corrected limb muscle mass, is used as an index of muscle mass^5, 42^. This study also used SMI as an index of muscle mass. The mean value of SMI in the participants was 6.3 ± 0.8 kg/m^2^. Sawaya et al.^43^ reported that the mean SMI by BIA method in female students was 6.1 ± 0.6 kg/m^2^, whereas Takahashi et al.^44^ reported that the mean SMI by BIA method was 6.30 ± 0.49 kg/m^2^. These were almost consistent with the present results.

Various studies reported on the cutoff values for sarcopenia using SMI. Walowski et al.^45^ reviewed 64 of these reports and concluded that each measurement is subject to random error and needs to be considered in terms of method, measurement device, and population reference. In addition, Walowski et al.^45^ recommend that the assessment of sarcopenia should be categorized by BMI, and then the SMI cutoff values should be set for each BMI category, rather than a uniform SMI cutoff value for each gender. World Health Organization (WHO) suggested that the cutoff point for BMI should be considered for each population^46^. For example, Asians are found to have a higher risk of type 2 diabetes and cardiovascular disease than Caucasians, even at a normal weight. However, the conclusion is that the international classification of BMI has not been redefined and the status quo has been maintained. The Japan Society for the Study of Obesity defines thinness and normal weight the same as the WHO classification and defines 25 or more as obesity rather than overweight^47^. Therefore, in considering sarcopenia, following the WHO international classification, for the time being, is reasonable. In addition, Walowski’s recommended that values are similar to those of the WHO international classification. Since this recommendation was attempted for Caucasians, comparing it with the present study results is difficult, which has a different population to refer to. Contrarily, in the present study, as the SMI group increased from low to high, the mean BMI in each group also increased. This result suggests that the recommendation of setting cutoff values for SMI by BMI category can be possibly applicable in Japan.

In the comparison of physical characteristics classified by the SMI categories, significant differences were found among the three groups by one-way ANOVA in all measurement items of physique. In addition, all measurement items, except for percentage of body fat, increased according to the increased SMI by trend analysis. Previous studies reported a positive correlation between muscle mass and bone mass^48–51^, which is consistent with the fact that bone mass increased with the increase in SMI in the present study. Low bone mass poses a risk of osteoporosis^52^. The elderly with sarcopenia have osteoporosis^53, 54^. As seen in the study results, a decreased muscle mass is considered to lead to a decreased bone mass and is complicated by osteoporosis because of the positive relationship between bone mass and muscle mass.

Increased body fat percentage indicates excess fat accumulation and is a risk of various non-communicable diseases. In the present results, an increased SMI was accompanied by an increased body fat mass, but not an increased body fat percentage. This means that an increased muscle mass is accompanied by an increased bone mass and body fat mass, resulting in increased body weight and BMI, but this is not necessarily a risk of non-communicable diseases.

Contrarily, regarding the association between the SMI and the physical fitness test, the higher the SMI, the higher the score in grip strength, upper body raising, repeated lateral jump, and vertical jump are. This indicates that increased muscle strength results in improved exercise performance. Therefore, regardless of muscle mass results from current exercise, a high volume of muscle mass affects exercise performance.

Daily exercise habits affect muscle mass or body fat mass. Previous studies reported that muscle mass is high in those who have a habit of exercising or are athletes^55, 56^, which indicates that sports increase muscle mass. Physical activity, especially during the growing phase, affects LBM and body fat percentage^57, 58^. In the present results, high SMI was significantly associated with a higher proportion of participants who liked exercise in childhood. Contrarily, no significant association was found among SMI and past exercise experience, current exercise habits, and current exercise preference. In addition, > 85% of participants in each SMI category do not currently have exercise habits. The fact that the proportion of young women who have a habit of regular exercise is small shows the same tendency as in previous studies^20, 24^. Therefore, the magnitude of SMI in the present subjects is possibly indirectly influenced by current exercise habits but reflects physical activity rather than those that are recognized as exercise.

The questionnaire survey found significant results in chi-square analysis and trend analysis regarding body perception, desired body shape, diet experience, and exercise preference in childhood among the High, Middle, and Low groups. The percentage of those who answered “obese” about body perception and the percentage of those who answered “become thin” showed a high value as the SMI increased. Previous studies also reported that the larger the BMI, the greater the proportion of people whobecome thin^33^. In this study, BMI showed a high value as the SMI increased. Therefore, the percentage of those who answered “obese” or “become thin” in the High group is considered high, which reflects its relationship with BMI.

A desire for thinness has been reported to be associated with diet behavior^20, 24, 33^. In the present participants, the proportion of those who had diet experience significantly increased as the SMI increased. No significant difference was found between the SMI categories as for the reason to desire thinness, but > 70% of participants in each SMI category cited “beauty” as the reason for becoming thin. In addition, no significant difference was found between the SMI categories; however, the main dieting method in each category was “diet restriction,” with a tendency for few people to go for “exercise or sports.” This result is consistent with previous study results on young women^20, 24^.

In addition, a desire for thinness and excessive dieting behavior pose a risk of eating disorder^59, 60^. Thus, EAT-26 was used to investigate the current eating behavior. Therefore, the higher the SMI is, the higher the value of the first factor “dieting” will be. Reports suggest the effect of physique on the total score of EAT-26 and “dieting”^61, 62^. From this, the high value of “Dieting” in the High group was considered to be influenced by the large physique. Therefore, to determine the relationship of the muscle mass to the body perception, desired body shape, and diet experience, an ANCOVA with body fat mass as a covariant was performed. As a result, muscle mass itself showed to be associated with body perception, desired body shape, and diet experience. A diet with only dietary restrictions without exercise has been reported to be promoted to reduce LBM^20^. In the present results, body weight and body fat mass increased as the SMI increased, but the body fat percentage did not significantly increase. Therefore, in the present participants, no particular rational reason was found to reduce body fat mass. In the present results, the higher the SMI, the higher the score of the physical fitness test is. In addition, muscle mass was reported to peak in the 20 s and gradually decrease with aging^10, 15^. Therefore, the higher the SMI, the higher the muscle mass should be maintained, and those who have high SMI should not lose weight.

There are several limitations to this study. First, the study sample was collected from a restricted geographic area in Japan, and the sample was relatively small. In future surveys, considering these factors is necessary for sampling female students in a wide area and try random sampling if possible. Contrarily, the average height, weight, and BMI in the present participants were 158.2 ± 5.2 cm, 54.2 ± 7.7 kg, and 21.6 ± 2.8, respectively. These values were almost similar with the national average of the same age (height, 158.2 ± 5.4 cm; weight, 52.4 ± 6.9 kg; BMI, 21.0 kg/m^2^)^63^. In addition, the mean value of the SMI in the present participants was 6.3 ± 0.8 kg/m^2^. Sawaya et al.^43^ reported that the mean SMI by BIA method in female students was 6.1 ± 0.6 kg/m^2^, whereas Takahashi et al.^44^ reported that the mean SMI by BIA method was 6.30 ± 0.49 kg/m^2^, which were almost consistent with the present results. Therefore, it is considered that participants in this study were less biased in terms of physique. Second, this study did not confirm exercise intensity or exercise amount; hence, it was not reflected. In the future, a more rigorous study should be considered by making a clear distinction between exercise intensity and amount. Third, some self-reported questionnaires used in the present study were insufficiently validated. Forth, the results of body composition or bone mineral contents were shown in the present, which was measured BIA. For seeking more accuracy, dual-energy x-ray absorptiometry (DXA) or magnetic resonance imaging (MRI) should be used. In addition, measuring students by carrying the DXA or MRI equipment to the school is difficult. Therefore, BIA methods were used in the present study. Fifth, for the purpose of exploring concerns about increasing proportions of underweight young women who have low muscle mass and increased long-term risk for sarcopenia, the present results cannot be immediately generalized, because the study population includes both underweight and normal weight women. In that case, it will be necessary to reconsider after increasing the number of underweight young women.

## Conclusions

This study examined the relationship between muscle mass and eating behavior, exercise habits, physical fitness, body shape recognition, desired body shape, and diet experience in female university students.

The more the skeletal muscle mass increased, the more the BMI, body fat mass, LBM, limb skeletal muscle mass, and whole-body bone mineral contents by trend analysis increased. The more the SMI, the higher the scores of physical fitness tests are. However, those who have high skeletal muscle mass perceived their body as “obese,” had a desire for thinness, and had diet experience. Those who had high skeletal muscle mass were high in the score of EAT-26 in dieting. ANCOVA, which was adjusted by body fat mass, revealed that those who had high skeletal muscle mass still perceived their body as obese, had a desire for thinness, and diet experience. These results indicate that the high mass of the body drives young females’ desire to be thin, whatever the body composition is, or even with exercise advantages.

Inherently, increased muscle mass is advantageous for physical activity. However, increased muscle mass can drive women’s desire to be thin, which increases the risk of muscle mass loss and potential diseases, such as sarcopenia. In addition, it has been reported that the woman's desire to be thin can lead to inappropriate weight loss behaviors^24, 60, 64^ and this can be improved with health education and health guidance^65–67^. Therefore, the results of this study indicate that health education and health guidance are necessary to prevent women’s desire to be thin to lead to inappropriate weight-loss behaviors since many women have a desire to be thin, especially young women.

## References

[1] Krebs D, Wong D, Jevsevar D, Riley PO, Hodge WA (1992). Trunk kinematics during locomotor activities. Phys. Ther..

[2] MacKinnon CD, Winter DA (1993). Control of whole body balance in the frontal plane during human walking. J. Biomech..

[3] Han SS (2010). Lean mass index: A better predictor of mortality than body mass index in elderly Asians. J. Am. Geriatr. Soc..

[4] Goodpaster BH (2008). Effects of physical activity on strength and skeletal muscle fat infiltration in older adults: A randomized controlled trial. J. Appl. Physiol..

[5] Cruz-Jentoft AJ (2010). Sarcopenia: European consensus on definition and diagnosis: Report of the European Working Group on Sarcopenia in Older People. Age Ageing.

[6] Rosenberg IH (1989). Summary comments: Epidemiological and methodological problems in determining nutritional status of older persons. Am. J. Clin. Nutr..

[7] Janssen I (2006). Influence of sarcopenia on the development of physical disability: The Cardiovascular Health Study. J. Am. Geriatr. Soc..

[8] Sanada K (2010). A cross-sectional study of sarcopenia in Japanese men and women: Reference values and association with cardiovascular risk factors. Eur. J. Appl. Physiol..

[9] Srikanthan P, Hevener AL, Karlamangla AS (2010). Sarcopenia exacerbates obesity-associated insulin resistance and dysglycemia: Findings from the National Health and Nutrition Examination Survey III. PLoS ONE.

[10] Janssen I, Heymsfield SB, Wang ZM, Ross R (2000). Skeletal muscle mass and distribution in 468 men and women aged 18–88 yr. J Appl Physiol..

[11] Yamada M, Moriguch Y, Mitani T, Aoyama T, Arai H (2014). Age-dependent changes in skeletal muscle mass and visceral fat area in Japanese adults from 40–79 years of age. Geriatr. Gerontol. Int..

[12] American College of Sports Medicine Position Stand (1998). Exercise and physical activity for older adults. Med Sci Sports Exerc..

[13] Karlsson MK, Nordqvist A, Karlsson C (2008). Physical activity, muscle function, falls and fractures. Food Nutr. Res..

[14] Peterson MD, Rhea MR, Sen A, Gordon PM (2010). Resistance exercise for muscular strength in older adults: A meta-analysis. Ageing Res. Rev..

[15] Gallagher D (1997). Appendicular skeletal muscle mass: Effects of age, gender, and ethnicity. J. Appl. Physiol..

[16] Takimoto H, Yoshiike N, Kaneda F, Yoshita K (2004). Thinness among young Japanese women. Am. J. Public Health..

[17] Ministry of Health, Labour and Welfare, Japan. Results of Year 2016 National Health and Nutrition Survey. https://www.mhlw.go.jp/content/000681180.pdf (2017) **(in Japanese)**

[18] Liu LK (2014). Sarcopenia, and its association with cardiometabolic and functional characteristics in Taiwan: Results from I-Lan Longitudinal Aging Study. Geriatr. Gerontol. Int..

[19] Yu R (2014). Incidence, reversibility, risk factors and the protective effect of high body mass index against sarcopenia in community-dwelling older Chinese adults. Geriatr. Gerontol. Int..

[20] Mase T (2012). Association between normal weight obesity and diet behaviors in female students. Nihon Koshu Eisei Zasshi..

[21] Deurenberg P, Deurenberg-Yap M, Guricci S (2002). Asians are different from Caucasians and from each other in their body mass index/body fat per cent relationship. Obes. Rev..

[22] Rush EC, Freitas I, Plank LD (2009). Body size, body composition and fat distribution: Comparative analysis of European, Maori, Pacific Island and Asian Indian adults. Br. J. Nutr..

[23] Wang J (1994). Asians have lower body mass index (BMI) but higher percent body fat than do whites: Comparisons of anthropometric measurements. Am. J. Clin. Nutr..

[24] Mase T (2013). Relationship of a desire of thinness and eating behavior among Japanese underweight female students. Eat Weight Disord..

[25] Ministry of Health, Labour and Welfare, Japan. Yearly report of long-term care insurance 2015. http://www.mhlw.go.jp/topics/kaigo/osirase/jigyo/15/index.html (2016) **(in Japanese)**

[26] Yuki A (2015). Epidemiology of sarcopenia in elderly Japanese. J. Phys. Fitness Sports Med..

[27] Yoshiko A (2017). Effect of 12-month resistance and endurance training on quality, quantity, and function of skeletal muscle in older adults requiring long-term care. Exp. Gerontol..

[28] Tanaka T (2021). A comparison of sarcopenia prevalence between former Tokyo 1964 Olympic athletes and general community-dwelling older adults. J. Cachexia Sarcopenia Muscle.

[29] Sayer AA (2008). The developmental origins of sarcopenia. J. Nutr. Health Aging.

[30] Begdache L, Patrissy CM (2021). Customization of diet may promote exercise and improve mental wellbeing in mature adults: The role of exercise as a mediator. J. Pers. Med..

[31] Monteiro LZ (2019). Weight status, physical activity and eating habits of young adults in Midwest Brazil. Public Health Nutr..

[32] Baumgartner RN (1998). Epidemiology of sarcopenia among the elderly in New Mexico. Am J Epidemiol..

[33] Mase T, Ohara K, Miyawaki C, Kouda K, Nakamura H (2015). Influences of peers’ and family members’ body shapes on perception of body image and desire for thinness in Japanese female students. Int. J. Womens Health..

[34] Mukai T, Crago M, Shisslak CM (1994). Eating attitudes and weight preoccupation among female high school students in Japan. J. Child Psychol. Psychiatry..

[35] Mukai T, Kambara A, Sasaki Y (1998). Body dissatisfaction, need for social approval, and eating disturbances among Japanese and American college women. Sex Roles.

[36] Garner DM, Olmsted MP, Bohr Y, Garfinkel PE (1982). The eating attitudes test: Psychometric features and clinical correlates. Psychol. Med..

[37] Ministry of Education, Culture, Sports, Science, and Technology: Implementation guidelines for the new physical fitness test. https://www.mext.go.jp/a_menu/sports/stamina/03040901.htm (in Japanese)

[38] Tyrovola JB, Odont XX (2015). The “Mechanostat theory” of frost and the OPG/RANKL/RANK system. J. Cell Biochem..

[39] Hamrick MW, McNeil PL, Patterson SL (2010). Role of muscle-derived growth factors in bone formation. J. Musculoskelet. Neuronal. Interact..

[40] Bikle DD (2015). Role of IGF-I signaling in muscle bone interactions. Bone.

[41] Kouda K (2019). Associations between serum levels of insulin-like growth factor-I and bone mineral acquisition in pubertal children: A3-year follow-up study in Hamamatsu, Japan. J. Physiol. Anthropol..

[42] Chen LK (2014). Sarcopenia in Asia: Consensus report of the Asian Working Group for Sarcopenia. J. Am. Med. Dir. Assoc..

[43] Sawaya Y (2018). Correlation between skeletal muscle mass index and parameters of respiratory function and muscle strength in young healthy adults according to gender. J. Phys. Ther. Sci..

[44] Takahashi H, Ishizaka M, Kubo A, Sadakiyo K, Suzuki Y (2017). Relationship between skeletal muscle mass and respiratory function of healthy adults. Rigakuryoho Kagaku..

[45] Walowski CO (2020). Reference values for skeletal muscle mass—Current concepts and methodological considerations. Nutrients.

[46] WHO Expert Consultation. Appropriate body-mass index for Asian populations and its implications for policy and intervention strategies. *Lancet.* **363**(9403), 157–163. 10.1016/S0140-6736(03)15268-3 (2004) 10.1016/S0140-6736(03)15268-3 14726171

[47] Lee JWR, Brancati FL, Yeh HC (2011). Trends in the prevalence of Type 2 Diabetes in Asians versus Whites: Results from the United States National Health Interview Survey, 1997–2008. Diabetes Care.

[48] Genaro PS, Pereira GA, Pinheiro MM, Szejnfeld VL, Martini LA (2010). Influence of body composition on bone mass in postmenopausal osteoporotic women. Arch. Gerontol. Geriatr..

[49] Pereira FB, Leite AF, Paula AP (2015). Relationship between pre-sarcopenia, sarcopenia and bone mineral density in elderly men. Arch. Endocrinol. Metab..

[50] Szulc P, Beck TJ, Marchand F, Delmas PD (2005). Low skeletal muscle mass is associated with poor structural parameters of bone and impaired balance in elderly men-the MINOS study. J. Bone Miner. Res..

[51] Verschueren S (2013). Sarcopenia and its relationship with bone mineral density in middle-aged and elderly European men. Osteoporos Int..

[52] Kanis JA (1994). Assessment of fracture risk and its application to screening for postmenopausal osteoporosis: synopsis of a WHO report. WHO Study Group. Osteoporos Int..

[53] Go SW, Cha YH, Lee JA, Park HS (2013). Association between sarcopenia, bone density, and health-related quality of life in Korean men. Korean J Fam Med..

[54] Sjöblom S (2013). Relationship between postmenopausal osteoporosis and the components of clinical sarcopenia. Maturitas.

[55] Abe T, Kawakami Y, Bemben MG, Fukunaga T (2011). Comparison of age-related, site-specific muscle loss between young and old active and inactive Japanese women. J Geriatr. Phys. Ther..

[56] Shitara K, Katsumata Y, Kumagawa D, Ikeda T, Hirano Y (2017). Age- and athletic event-related differences in trunk muscularity in junior athletes: A comparison with the results for the senior athletes. Jpn. J. Phys. Fitness Sports Med..

[57] Abbott RA, Davies PS (2004). Habitual physical activity and physical activity intensity: Their relation to body composition in 5.0–10.5-y-old children. Eur. J. Clin. Nutr..

[58] Baxter-Jones AD, Eisenmann JC, Mirwald RL, Faulkner RA, Bailey DA (2008). The influence of physical activity on lean mass accrual during adolescence: A longitudinal analysis. J. Appl. Physiol..

[59] Patton GC, Selzer R, Coffey C, Carlin JB, Wolfe R (1999). Onset of adolescent eating disorders: population based cohort study over 3 years. BMJ.

[60] Muro-Sans P, Amador-Campos JA (2007). Prevalence of eating disorders in a Spanish community adolescent sample. Eat Weight Disord..

[61] Yang SJ, Kim JM, Yoon JS (2010). Disturbed eating attitudes and behaviors in South Korean boys and girls: A school-based cross-sectional study. Yonsei Med. J..

[62] Yannakoulia M (2004). Disordered eating attitudes: An emerging health problem among Mediterranean adolescents. Eat Weight Disord..

[63] Japan Sports Agency. The report of FY 2016 survey on physical fitness, athletic performance. https://www.mext.go.jp/sports/b_menu/toukei/chousa04/tairyoku/kekka/k_detail/1396900.htm (2017).

[64] Ko N, Tam DM, Viet NK, Scheib P, Wirsching M, Zeeck A (2015). Disordered eating behaviors in university students in Hanoi, Vietnam. J. Eat Disord..

[65] Yager Z, O'Dea J (2010). A controlled intervention to promote a healthy body image, reduce eating disorder risk and prevent excessive exercise among trainee health education and physical education teachers. Health Educ. Res..

[66] Blackstone SR, Sangiorgio C, Johnson AK (2020). Peer recognition of disordered eating behaviors: Implications for improving awareness through health education. Am. J. Health Educ..

[67] Pursey KM, Burrows TL, Barker D, Hart M, Paxton SJ (2021). Disordered eating, body image concerns, and weight control behaviors in primary school aged children: A systematic review and meta-analysis of universal–selective prevention interventions. Int J Eat Disord..

